# Multi-Model Segmentation Algorithm for Rotator Cuff Injury Based on MRI Images

**DOI:** 10.3390/bioengineering12030218

**Published:** 2025-02-21

**Authors:** Mengqi Li, Jingchao Fang, Haonan Hou, Li Yuan, Jin Guo, Zhenlong Liu

**Affiliations:** 1 Department of Sports Medicine, Peking University Third Hospital, Institute of Sports Medicine of Peking University, Beijing 100191, China; mengqi_li@icloud.com; 2 School of Automation and Electrical Engineering, University of Science and Technology Beijing, Beijing 100083, China; m202220708@xs.ustb.edu.cn (H.H.); lyuan@ustb.edu.cn (L.Y.); 3 Department of Radiology, Peking University Third Hospital, Beijing 100191, China; fangzzxxcc@163.com; 4 Key Laboratory of Knowledge Automation for Industrial Processes, Ministry of Education, Beijing 100083, China; 5 Beijing Key Laboratory of Sports Injuries, Beijing 100191, China; 6 Engineering Research Center of Sports Trauma Treatment Technology and Devices, Ministry of Education, Beijing 100191, China

**Keywords:** MRI, rotator cuff injury, deep learning, AI segmentation, multi-model mechanism

## Abstract

This paper proposes an AI-based diagnostic method using MRI images for rotator cuff injuries to assist in treatment by segmenting tear areas and assessing tear severity. A multi-model deep learning network based on Unet + FPN architecture was developed to automatically segment rotator cuff injury images and determine tear grades. A dataset of 376 patients with 5640 images was used for training, with an additional 94 patients and 1410 images reserved for testing. To optimize segmentation, a tailored matching strategy was applied, achieving an Intersection over Union (IoU) of 0.79 ± 0.01 and a Dice coefficient of 0.75 ± 0.01, indicating high accuracy in segmenting tear areas. For tear severity indicators, the accuracy of estimating retraction (ER) reached 0.92 ± 0.02, and the accuracy of estimating stop tear width (ESTW) reached 0.79 ± 0.01. As the first AI algorithm specifically developed for diagnosing rotator cuff injuries, this platform demonstrates promising accuracy in both tear segmentation and severity assessment, aiming to support doctors in providing efficient, accurate diagnoses of rotator cuff tears.

## 1. Introduction

Rotator cuff tears account for 5% to 40% of shoulder disorders [1]. Because of the lack of understanding of rotator cuff tears, they are easily misdiagnosed as diseases such as frozen shoulder, and their misdiagnosis rate is high. Once misdiagnosed, the treatment is delayed and in severe cases, the shoulder joint function can be lost. MRI is the preferred imaging modality for diagnosing tears, demonstrating improved diagnostic accuracy compared to previous methods [2]. Because of the uneven level of orthopaedic and sports medicine treatment, there are difficulties in diagnosing rotator cuff tears when reading MRI of the shoulder [3]. In order to assist doctors in diagnosing rotator cuff tears, this paper constructed an artificial intelligence diagnostic platform (Rotator Cuff Tear Diagnostic Platform, RCTDP) to efficiently and rapidly achieve diagnosis and grading of rotator cuff tears and reduce the occurrence of misdiagnosis and missed diagnoses.

**Figure 1:**
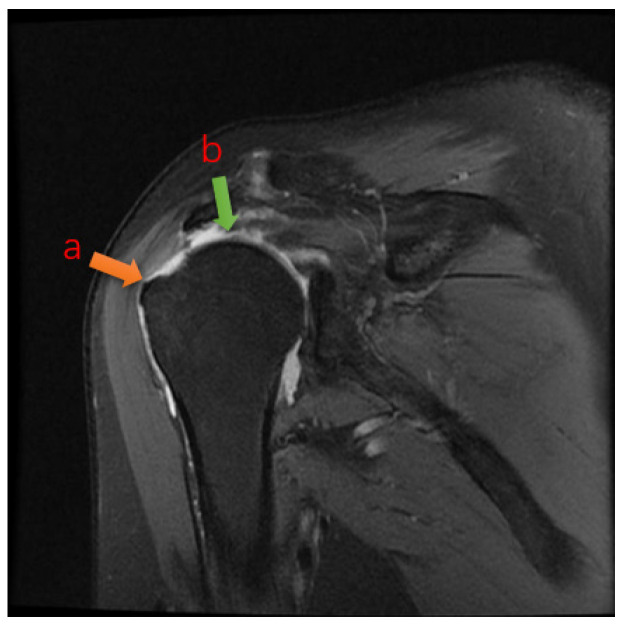
A typical tear image of Ocor T2. The arrow shows that the tendon is retracted from point a to point b after rupture, and the joint fluid-filled area is visible in the highlighted area between point a and point b. The joint fluid-filled area is the tear region.

In the clinical diagnosis of rotator cuff tears nowadays, doctors often need to take a close look at multiple MRIs of the shoulder and combine them into a coherent sequence of MRIs to determine whether the area of the lesion is an effusion or a tear [4], and to measure the length of the tear with calipers to give the grade of the tear (Figure 1). The AI diagnostic method proposed in this paper can assist doctors to greatly improve the diagnostic efficiency, which has many advantages over traditional clinical diagnosis.

Current research in deep learning for image segmentation focuses on the differentiation of common household items, most of which have obvious and unique visual features, making segmentation relatively simple. In the field of medical images, specific segmentation objects are mainly focused on brain MRI and lung CT; these images often also have obvious and regular lesion features. However, the damaged areas of rotator cuff tears are of different sizes and irregular shapes, so the existing segmentation network model of medical images cannot perform satisfactorily on these lesions.

Current research for intelligent diagnosis of medical images is mainly focused on segmentation or categorization of lung CT and brain MRI lesions, and very few diagnoses of rotator cuff tears have been reported. In addition, mature intelligent algorithms, such as Unet and DeeplabV3, can only segment elliptical and rectangular lesion features, and they are unable to do anything with a rotator cuff tear, which occupies a very small area and has an irregular shape. Therefore, there is an urgent need to propose a deep network model for intelligent diagnosis of this lesion feature, which can assist the doctors in realizing a more efficient workflow.

The purpose of this paper was to develop an artificial intelligence diagnostic method for rotator cuff injury based on MRI images, which enabled the computer to accurately segment the tear area on the patient’s serial images and automatically give a judgment of the patient’s tear degree, assisting the relevant doctors in the diagnosis and treatment of this condition.

## 2. Methods

### 2.1. Study Design and Patients

The MRI data for this study were obtained from the author’s institute between 2021 and 2022, and a total of 470 patients were included. All patient data were privacy-protected to ensure that the experiments were conducted in accordance with the relevant ethical norms. To ensure the stability of the algorithm output, the order of patients was randomized, and 376 patient datasets were selected as training subjects for image screening and labeling, and the remaining 94 patient datasets were used as the testing set for the final algorithm validation.

### 2.2. Image Acquisition: Ocor T2 fs Sequence

The data used in this study were MRI scan sequence images of patients (device GE HealthCare, 3.0T MR750 WIDE, GE HealthCare, Beijing, China). Each sequence consisted of 15~20 2D images. The size of the images was 320 × 224 (pixel), the field of view (FOV) was 16 × 16 (cm), the thickness was 3 mm, and the interval between images was 0.5 mm [5]. For each patient, it is often necessary to take multiple sequences because of the problem of moving during image acquisition, from which the most clearly imaged set of sequences was then selected as the data for training. Figure 1 shows examples of the workflow.

Details of the Ocor T2 fs Sequence can be seen in the Supplementary Materials.

### 2.3. Data Processing and Labeling

The format conversion of the images needs to be completed in advance for the training and test sets after random assignment. Because DICOM format files cannot be directly sampled, convolved, or have other network training steps applied, they need to be converted to PNG format images and then input to the deep learning network as Tensor [6]. The resolution of the image after conversion to Tensor is 512 × 512 (pixel), and the color value of each pixel ranges from 0~255.

The training set images converted to PNG format were manually labeled to form a labeled dataset. Labelme software (V5.4.1) was used to implement the labeling function, which is written in Python (3.9) and Qt5 (5.15). The trained doctor outlines the tear area through a curve within the software, and once the closure of the loop is completed, the software automatically generates a label map, with the area of the surrounded tear site covered in red and the peripheral normal part fully covered in black [7], with a clear difference in the color values between the two, which can clearly distinguish the tear area. Because the width of the outline is only 3 pixels, the impact on the later network training and segmentation comparison is negligible.

Each image in the training and test sets was individually labeled by two expert doctors, a senior specialist in sports medicine and an experienced radiologist with a high level of experience with rotator cuff injury diagnosis; both could accurately label the images to ensure the accuracy and validity of the labels.

### 2.4. Neural Network Design and Training

Compared to the intelligent diagnosis of skin cancer, the rotator cuff tear lesions targeted in this paper vary in size and shape and do not have obvious regular features in their edge contour, so the existing medical image depth models cannot learn the features of the disease for diagnosis [8]. By observing a large number of MRI images of rotator cuff injuries, it can be found that the tear region accounts for a relatively small percentage of the whole image, about 4%, while the tear shape varies from sequence to sequence for the same patient and the tear region often varies greatly from patient to patient [9], so the traditional CNN method is not applicable to rotator cuff injury segmentation, and a new deep learning network needs to be designed for the region of the site for detection and segmentation.

**Figure 2:**
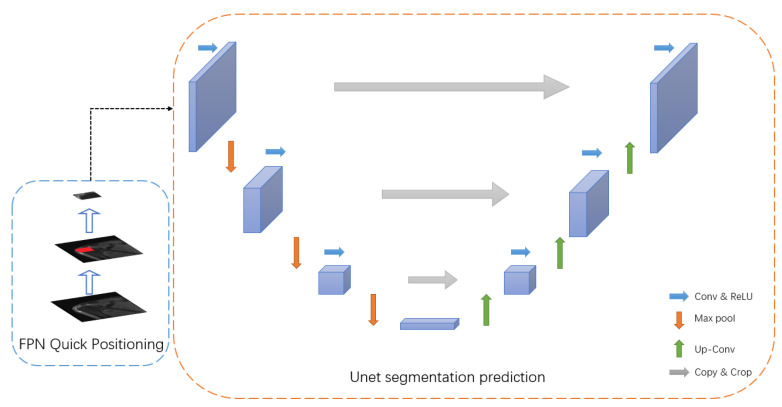
The FPN + Unet architecture.

In order to make the network output more accurate and the segmentation to have better robustness and a shorter output time, this work constructed the Unet + FPN network architecture (Figure 2), where Unet is a classic network architecture for medical image segmentation [10] and FPN is an efficient architecture for tiny object detection [11]; their specific design and built-in parameters are detailed in the Supplementary Materials. Overall, the network architecture proposed in this paper is a two-dimensional convolutional network with a total of three channels of RGB for the input and two channels for the output to display the corresponding segmentation results, and the specific display is shown in the Supplementary Materials.

Because each patient’s rotator cuff labeling sequence presents an anterior to posterior sequence, it generally exhibits an anterior-middle-posterior triadic structure [12]. The anterior and posterior segments tend to have extremely small tear areas and visually resemble clusters of dots composed of very few vegetative dots; the middle segment has larger tear areas and the shape of the anterior–posterior sequence tends to be continuous with little contour variation. Because the size and shape of the front, back and middle segments are extremely different, the differences among them are expressed as features to be learned by deep networks. It is necessary to design three-layered networks to learn the features of the sequence images with different intervals so as to achieve fast and accurate segmentation, and the details of the specific parameters, etc. are shown in the Supplementary Materials.

**Figure 3:**
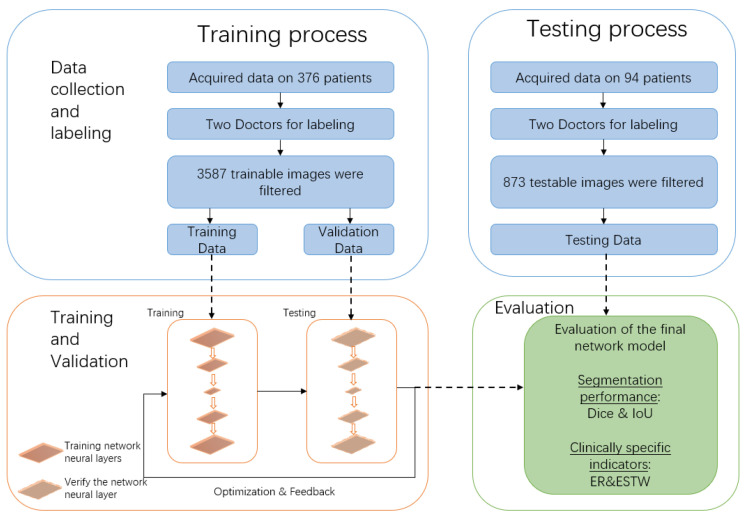
The workflow of the learning process and final evaluation of the deep network.

The network was trained for 80 epochs, and a weighted Adam optimizer was used to optimize the loss function. The initial learning rate was set to 0.01, and the decay rate was set to 0.0001. The purpose of the network training was to optimize parameters to minimize the mean square error of the loss function. The flow chart of the training and testing can be seen in Figure 3.

Details about the network architecture can be seen in the relevant content.

### 2.5. Automatic Segmentation of Predictions

**Figure 4:**
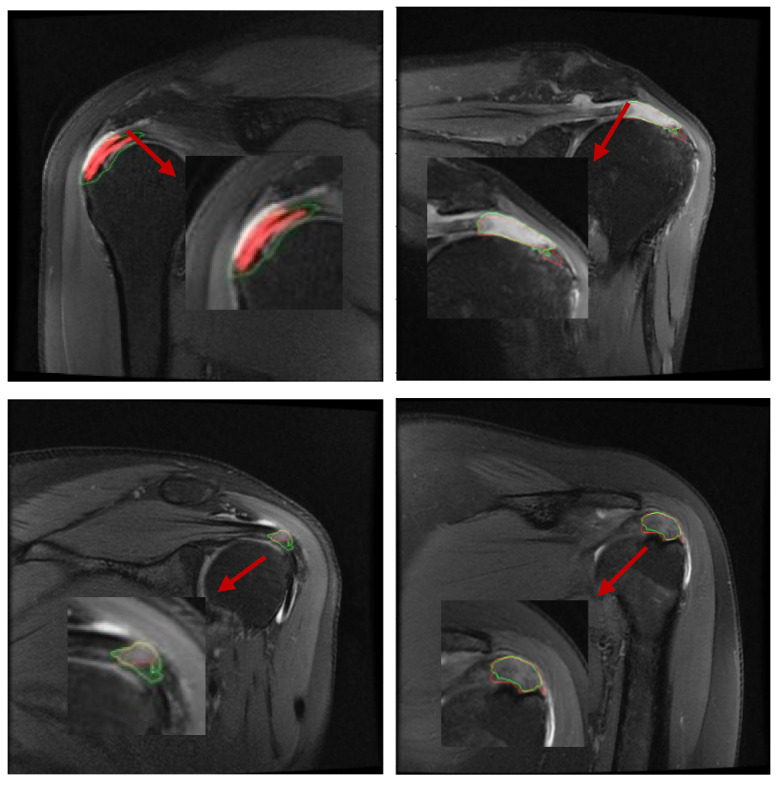
Comparison between the physician-generated labels and the AI algorithm’s predicted segmentation results. The red regions represent manual segmentations by doctors, while the green contour outlines the AI’s predicted segmentation.

Because the output of the deep neural network is two-channel, i.e., the output pixel points are predictively classified and assigned color values, it is possible to distinguish torn regions from normal regions by the difference in color values of the adjacent points [13]. Through continuous feedback learning, the deep learning network will give classification results for both color values, which can be evident within the software to see the segmented regions and boundaries (Figure 4), and thus be compared with the labels to calculate the relevant segmentation metrics [14]. In summary, the overall process of the segmentation algorithm is to first determine the approximate location of the torn region in the full image by the localization detection algorithm, and then to segment the torn region by classifying the pixel points into two categories, torn and normal, by operations such as convolution sampling classification.

Details about the network outputs can be seen in the relevant content.

### 2.6. Model Deployment and Integration

The network model has been trained to fix the built-in parameters and retain only the prediction estimation function, and the overall Python code has been encapsulated into the program software. The computer software program, built in Java, has not only integrated the aforementioned segmentation algorithm functions but has also designed an aesthetically pleasing UI interface to achieve a graphical presentation for the users, including a dynamic visualization window, buttons for each implemented function call, and a menu for each process switch. The physician is able to observe the original MRI image of the rotator cuff, the segmented tear area along with its dimensions, and the tear grade judgment provided by the algorithm by simply clicking with the mouse, thereby ensuring that all the diagnostic work has been performed within the software. The specific display is shown in the Supplementary Materials.

### 2.7. Statistical Analysis

In order to evaluate the effectiveness of the segmentation model for rotator cuff injury regions and to compare it with segmentation results by experts, a performance index function was used and designed to evaluate the performance of the segmentation algorithm under different clinical perspectives. Thus, a baseline was set to determine whether the performance of the model meets the requirements to be used in a clinical setting.

Accuracy is usually a performance indicator to measure whether the classification network can correctly categorize samples [15], because the research task of this paper is not only the accuracy of statistical classification, but also the location relationship of output pixel points, and it is necessary to introduce new evaluation metrics on this basis as an important aspect of measuring the performance of the segmentation networks.

Two mathematical metrics, IoU and the Dice coefficient (where a high Dice coefficient indicates a high level of similarity between the predicted and ground truth), are introduced here. Intersection over Union (IoU) is a measure of the accuracy of detecting the corresponding object in a given dataset [16] and can be used for any task that yields a prediction range (bounding boxes) in the output, which is defined as follows:

$$IoU\left(X,Y\right)=\frac{\left|\left|X⋂Y\right|\right|}{\left|\left|X⋃Y\right|\right|}$$

The Dice coefficient is a set similarity measure function [17] that also measures the relevant overlap and is defined as follows: X and Y, i.e., the greater the approximation of the segmentation region; being closer to 0 means a smaller overlap between the two.

In this paper, two unique validation indexes were designed based on clinical needs for rotator cuff clinical diagnosis, namely, the accuracy of segmentation region retraction estimation and the accuracy of stop tear distance estimation.

We denote the segmented region retraction estimation error as $E_{h}$, which is defined as follows:

$$E_{h}=\frac{\left|L_{r}-L_{e}\right|}{L_{e}}$$

where $L_{r}$ denotes the actual retraction of the tear region, $L_{e}$ is the estimated retraction of the tear region, and the retraction is defined as follows:

$$L=3.3n-0.3\left(mm\right)$$

We denote the estimation error of the stop tear distance as $E_{l}$, which is defined as follows:

$$E_{l}=\frac{\left|l_{r}-l_{e}\right|}{l_{r}}$$

where $l_{r}$ indicates the length of the rectangular box in the actual tear region of the layer image and $l_{e}$ indicates the length of the rectangular box in the estimated tear region. Both of these targeted metrics can reflect the performance of the segmentation algorithm in a clinical sense.

## 3. Results

### 3.1. Segmentation Performance

Because doctors do not calculate the size of specific tear regions for the diagnosis of rotator cuff tears in the actual clinical setting, the IoU and Dice coefficients are used here only to assess the accuracy for segmentation and calculation of tear regions [18,19]. There is no previous report of AI diagnosis of tears in the rotator cuff field, so there is no direct object of comparison in this paper, and the corresponding index results are given here directly.

The mIoU (average IoU) of the test set reached 0.79, where specifically for each image, the IoU of the middle image can reach 0.9, and the IoU results of the front and back end images will be low because the tear area is irregular in size and shape. The average Dice coefficient of the test set reached 0.75, with the same phenomenon of small at both ends and high in the middle.

For the clinically designed special metrics, the accuracy of the estimation of the stop tear distance can reach 0.92, showing that the algorithm we designed is closer to the doctor’s judgment in estimating the stop tear distance of a rotator cuff tear. For the estimation performance of the segmented region retraction, an accuracy of 0.8 was achieved, indicating that the estimation of the number of tear image layers for the same patient was also close to the doctor’s diagnosis. Compared with the two mathematical metrics of IoU and the Dice coefficient, the performance of the above two clinical metrics is closer to the actual usage scenario, showing the accuracy and reliability of this algorithm.

### 3.2. Segmentation Performance Using Unet Alone

In order to compare it with the segmentation algorithm proposed in this paper, the classical Unet was used here for training and observing its segmentation effect.

Because of the lack of fast localization of the tear region by FPN and the difficulty of fully learning complex full-segment sequences as a single framework network, more than one tear region appears in the traditional Unet output image, and this predicted segmentation result is incompatible with the clinical requirements and is no longer meaningful for analyzing the relevant assessment metrics. This method indicates that the traditional network architecture is no longer suitable for segmentation diagnosis of complex lesions like rotator cuff tears, and complex deep networks incorporating multi-level structures need to be constructed to address the needs.

### 3.3. Summary

In this paper, a complex deep network model based on FPN + Unet was proposed for fast localization and accurate segmentation of rotator cuff tear, and the performance of the algorithm reached the optimal level of current algorithms in both common segmentation metrics and clinical metrics.

## 4. Discussion

In this paper, we propose an AI segmentation algorithm of the tear region for rotator cuff MRI images, which can accurately give the grading of the degree of tear and is expected to assist doctors in rapidly diagnosing rotator cuff tears and reducing the rates of misdiagnosis and underdiagnosis.

The object of this paper is the torn rotator cuff area, and there is no AI diagnosis and treatment study for this lesion in the industry. Similar works on artificial intelligence diagnosis and treatment of the shoulder have focused on CT-based determination of shoulder bones and other pathologies. To address the current situation that there is no correlation, this paper innovatively constructed a targeted segmental fusion deep network to learn the features of the tear region based on MRI image sequences. The deep learning network can learn the feature information of the torn lesion by simulating the process of determining the size of the torn area in the clinical setting. The network model learns repeatedly and then generates a predicted mask of the input image to compare with the doctor’s judgment, which is difficult for traditional image segmentation networks to perform.

In order to compare it with the segmentation algorithm proposed in this paper, the classical Unet network was used here for training and to observe its segmentation effect. Because of the lack of fast localization of the tear region by FPN and the difficulty of fully learning complex full-segment sequences as a single framework network, more than one tear region appears in the traditional Unet output image, and this predicted segmentation result is seriously incompatible with the clinical requirements and is no longer meaningful for analyzing the relevant assessment metrics. These results indicate that the traditional network architecture is no longer suitable for segmentation diagnosis of complex lesions like rotator cuff tears, and complex deep networks incorporating multi-level structures need to be constructed to address the clinical needs.

Compared with the intelligent segmentation research for tuberculosis, brain tumors, cancer, etc., the difficulty of the research content in this paper is that the region of the lesion is irregular in size, and there is no obvious rule for the shape of the contour, so the existing generalized networks, such as Unet and DeeplabV3, are unable to carry out the segmentation of this lesion efficiently and can only be applied to the ellipsoid and rectangle-like features of the lesion.

Since only coronal sequences are currently used, later on, we will consider adding sagittal and transverse axial sequences or 3D sequences for multi-dimensional sequence diagnosis to obtain more accurate diagnostic results.

According to the output display and the related indexes of the test, the diagnostic level of the algorithm has reached a very high level, and because the research on AI diagnosis of shoulder diseases is only in the initial stage, this method can be transferred to other disease diagnosis work, which has a wider development prospect and greater value of practical application.

## 5. Conclusions

In this paper, we present a novel artificial intelligence diagnostic platform for accurately segmenting and assessing rotator cuff injuries using MRI images. The proposed method, which integrates a multi-model deep learning network based on Unet + FPN architecture, demonstrates significant promise in both segmentation precision and diagnostic accuracy for rotator cuff tear grading. The findings suggest that this AI-driven approach holds substantial potential to support doctors in delivering efficient, accurate diagnoses for rotator cuff injuries, potentially reducing diagnostic time and improving patient outcomes.

## Data Availability

Data will be made available on reasonable requests.

## Supplementary Materials

The following supporting information can be downloaded at: https://www.mdpi.com/article/10.3390/bioengineering12030218/s1, Figure S1: Rectangular frame diagram; Table S1: Results across the different neural network architectures assessed in terms of Dice coefficient and clinical parameters. References [10,20,21,22,23,24,25,26,27,28] are cited in the supplementary materials.
