# Supplementary material for: Multi-Model Segmentation Algorithm for Rotator Cuff Injury Based on MRI Images
Source: Bioengineering (Basel). 2025 Feb 21;12(3):218. doi: 10.3390/bioengineering12030218 (PMC11939712; doi:10.3390/bioengineering12030218)
Supplement: Supplementary file 1 [file bioengineering-12-00218-s001.zip › bioengineering-3449273-supplementary.pdf]

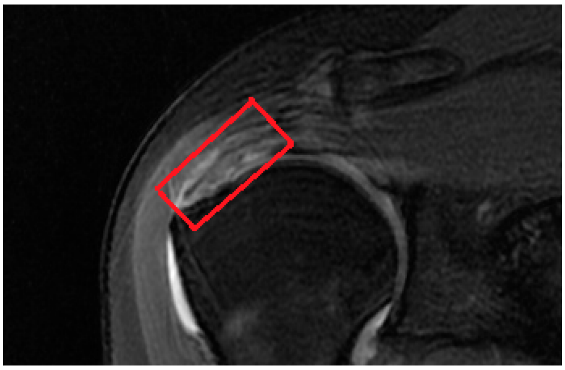

Figure S1. Rectangular frame diagram.

Table S1. Results across the different neural network architectures assessed in terms of Dice coefficient and clinical parameters.

Network			Dice Coefficient	mIoU	HE	LE
Architecture	Method	Backbone	vs. E1<br>vs. E2	vs. E1<br>vs. E2	vs. E1<br>vs. E2	vs. E1<br>vs. E2
Unet+FPN*	Boundary	EfficientNetB3	0.79	0.75	0.79	0.92
UNet	Semantic Segmentation	Standard	0.61	0.51	0.75	0.84
UNet	Semantic Segmentation	EfficientNetB3	0.67	0.53	0.75	0.84
UNet++	Semantic Segmentation	EfficientNetB3	0.75	0.61	0.76	0.85
DeepLabV3	Semantic Segmentation	EfficientNetB3	0.62	0.52	0.74	0.82

Supplementary References

10. Ronneberger, O.; Fischer, P.; Brox, T. U-net: Convolutional networks for biomedical image segmentation. In *Medical Image Computing and Computer-Assisted Intervention—MICCAI 2015: 18th International Conference, Munich, Germany, 5–9 October 2015*; Proceedings, Part III 18; Springer International Publishing: Cham, Switzerland, 2015.
20. Blansit, K.; Retson, T.; Masutani, E.; Bahrami, N.; Hsiao, A. Deep Learning-based Prescription of Cardiac MRI Planes. *Radiol. Artif. Intell.* **2019**, *1*, e180069.
21. Uysal, F.; Hardalaç, F.; Peker, O.; Tolunay, T.; Tokgöz, N. Classification of Fracture and Normal Shoulder Bone X-Ray Images Using Ensemble and Transfer Learning With Deep Learning Models Based on Convolutional Neural Networks. *arXiv* **2021**, arXiv:2102.00515.
22. Le, W.T.; Maleki, F.; Romero, F.P.; Forghani, R.; Kadoury, S. Overview of Machine Learning: Part 2: Deep Learning for Medical Image Analysis. *Neuroimaging Clin. North Am.* **2020**, *30*, 417–431. https://doi.org/10.1016/j.nic.2020.06.003.
23. He, K.; Gkioxari, G.; Dollár, P.; Girshick, R. Mask R-CNN. In Proceedings of the 2017 IEEE International Conference on Computer Vision (ICCV), Venice, Italy, 22–29 October 2017; IEEE: Piscataway, NJ, USA, 2017.
24. MacDermid, J.C.; Bryant, D.; Holtby, R.; Razmjou, H.; Faber, K.; JOINTS Canada; Balyk, R.; Boorman, R.; Sheps, D.; McCormack, R.; et al. Arthroscopic Versus Mini-open Rotator Cuff Repair: A Randomized Trial and Meta-analysis. *Am. J. Sports Med.* **2021**, *49*, 3184–3195.
25. Albumentations-Team. Albumentations. Available online: https://github.com/albumentations-team/albumentations (accessed on 14 June 2022).
26. Guan, B.; Zhang, G.; Yao, J.; Wang XWang, M. Arm fracture detection in X-rays based on improved deep convolutional neural network. *Comput. Electr. Eng.* **2020**, *81*, 106530.
27. van der Walt, S.; Schönberger, J.L.; Nunez-Iglesias, J.; Boulogne, F.; Warner, J.D.; Yager, N.; Gouillart, E.; Yu, T. scikit-image: Image processing in Python. *PeerJ* **2014**, *2*, e453.

28. GitHub-qubvel/segmentation_models.pytorch: Segmentation Models with Pretrained Backbones. PyTorch. Available online: https://github.com/qubvel/segmentation_models.pytorch (accessed on 23 November 2021).
